# Progress Toward Global Eradication of Dracunculiasis — January 2013–June 2014

**Published:** 2014-11-21

**Authors:** Donald R. Hopkins, Ernesto Ruiz-Tiben, Mark L. Eberhard, Sharon L. Roy

**Affiliations:** 1 The Carter Center, Atlanta, Georgia; 2 Division of Parasitic Diseases and Malaria, Center for Global Health, CDC; 3 Division of Foodborne, Waterborne, and Environmental Diseases, National Center for Emerging and Zoonotic Infectious Diseases and World Health Organization Collaborating Center for Research, Training, and Eradication of Dracunculiasis, CDC

Dracunculiasis (Guinea worm disease) is caused by *Dracunculus medinensis*, a parasitic worm. Approximately 1 year after a person acquires infection from contaminated drinking water, the worm will emerge through the skin, usually on the lower limb. Pain and secondary bacterial infection can cause temporary or permanent disability that disrupts work and schooling. In 1986, the World Health Assembly called for dracunculiasis elimination (1). The global Guinea Worm Eradication Program, supported by The Carter Center, World Health Organization (WHO), UNICEF, CDC, and other partners, began assisting ministries of health of countries in which dracunculiasis is endemic in meeting this goal. At that time, an estimated 3.5 million cases occurred each year in 20 countries in Africa and Asia (1,2). This report updates published (3–5) and unpublished surveillance data reported by ministries of health and describes progress toward dracunculiasis eradication. A total of 148 cases were reported in 2013 from five countries (in order of prevalence: South Sudan, Chad, Mali, Ethiopia, and Sudan) compared with 542 cases in 2012 from four countries (South Sudan, Chad, Mali, and Ethiopia). The disease remains endemic in four countries in 2014 (South Sudan, Chad, Mali, and Ethiopia), but the overall incidence is falling faster in 2013 compared with 2012 (by 73%) and continues to fall faster in the first 6 months of 2014 (by 71%) compared with the same period in 2013. Failures in surveillance and containment, lack of clean drinking water, insecurity in Mali and parts of South Sudan, and an unusual epidemiologic pattern in Chad (6) are the main remaining challenges to dracunculiasis eradication.

Because the lifecycle of *D. medinensis* is complex, its transmission can be interrupted using several strategies (4). Dracunculiasis can be prevented with four main interventions: 1) educating residents in communities where it is endemic, and particularly persons from whom worms are emerging, to avoid immersing affected body parts in sources of drinking water, 2) filtering potentially contaminated drinking water through a cloth filter, 3) treating potentially contaminated surface water with the insecticide temephos (Abate), and 4) providing safe drinking water from bore-hole or hand-dug wells (7). Containment of transmission,* is achieved through four complementary measures: 1) voluntary isolation of each patient to prevent contamination of drinking water sources, 2) provision of first aid, 3) manual extraction of the worm, and 4) application of occlusive bandages.

Countries enter the WHO precertification stage of eradication after completing 1 full year without reporting any indigenous† cases (*D. medinensis* has approximately a 1-year incubation period [range = 10–14 months]) (7). A case of dracunculiasis is defined as infection occurring in a person exhibiting a skin lesion or lesions with emergence of one or more Guinea worms. Each infection is counted as a case only once during a calendar year. An imported case is an infection resulting from ingestion of contaminated water from a source identified through patient interviews and epidemiologic investigation in a place (i.e., another country or village within the same country) other than in the community where the patient is detected and reported. Three countries where transmission of dracunculiasis was previously endemic (Ghana, Kenya, and Sudan) are in the precertification stage of eradication.

In each country affected by dracunculiasis, a national eradication program receives monthly reports of cases from each village that has endemic transmission. Reporting rates are calculated by dividing the number of villages with endemic dracunculiasis that report each month by the total number of villages with endemic disease. All villages with endemic dracunculiasis are kept under active surveillance, with daily searches of households for persons with signs and symptoms suggestive of dracunculiasis. These searches are conducted to ensure that detection occurs within 24 hours of worm emergence so that patient management can begin to prevent contamination of water sources. Villages in which endemic transmission of dracunculiasis is interrupted (i.e., zero cases reported for ≥12 consecutive months) also are kept under active surveillance for 3 consecutive years.

WHO certifies a country free from dracunculiasis after that country maintains adequate nationwide surveillance for ≥3 consecutive years and demonstrates that no cases of indigenous dracunculiasis occurred during that period. As of the end of 2013, WHO had certified 197 countries, areas, and territories as free from dracunculiasis (3). Nine countries remain to be certified: four countries where it is currently endemic (South Sudan, Chad, Mali, and Ethiopia), three countries in the precertification stage (Ghana, Kenya, and Sudan), and two countries never known to have had endemic dracunculiasis (Angola and the Democratic Republic of the Congo).

Although the 1991 and 2004 World Health Assembly goals to eradicate dracunculiasis globally in 1995 and 2009, respectively, were not achieved (7,8), considerable progress toward eradication has been made since 1986 in reducing the annual number of reported cases. This progress continued with a 73% decrease in cases between 2012 (542 cases) and 2013 (148) followed by a 71% decrease in cases during the first 6 months of 2014 (27) compared with the same period in 2013 (92). This 71% decrease in cases during the first half of 2014 compared with the same period in 2013 did include an increase in cases in Chad (from five cases in 2013 to six cases in 2014), but cases decreased in Ethiopia, Mali, and South Sudan. There also was a 29% reduction in the number of villages in these four countries reporting cases during January–June 2014 (20 villages) compared with January–June 2013 (28).

Surveillance is a challenge everywhere dracunculiasis exists. Of particular concern, surveillance for dracunculiasis remains constrained in most dracunculiasis-affected areas of Mali because of insecurity since March 2012. CDC tested 50 specimens during January 2013–June 2014 from suspected cases in humans in seven countries in which dracunculiais is or was endemic; 22 (44%) specimens were determined to be *D. medinensis*.

## Country Reports

### South Sudan

The 10 southern states of the former Sudan became the independent Republic of South Sudan in July 2011. The South Sudan area reported all of the indigenous cases since 2002, except for three cases detected in Sudan in 2013. The South Sudan Guinea Worm Eradication Program reported 113 cases in 2013, of which 76 (67%) were contained (Table 1), which was a reduction of 78% from the 521 cases reported in 2012. During January–June 2014, the South Sudan Guinea Worm Eradication Program reported a provisional total of 19 cases (79% contained), from 13 villages, compared with 74 cases (70% contained) reported from 52 villages in January–June 2013; a reduction of 74% in cases and 75% in the number of villages reporting cases (Table 2). South Sudan reported zero cases during November 2013–February 2014. Of the cases reported in the first 6 months of 2014, 95% were from Kapoeta East County (in Eastern Equatoria State), where failure to repair a key bridge that collapsed in May 2012 made delivery of supplies more complicated and costly. As previously described (4), movements of persons along multiple routes for seasonal activities such as livestock grazing and farming as well as sporadic insecurity created during interethnic cattle raiding and other reasons have presented unusually complex challenges to this program.

A severe political crisis in December 2013 disrupted program operations when all expatriate staff assisting the program were evacuated from the country for several weeks, although most national staff continued to work in the areas with highest prevalence. However, the coverage with interventions remains high despite the challenges (Table 1). In April 2014, the commissioner of Health of Eastern Equatoria State personally launched South Sudan’s cash reward for reporting a confirmed case of dracunculiasis (500 South Sudanese pounds, or about US $125), during a 10-day tour of dracunculiasis-affected villages.

### Chad

After a decade with no reported cases, Chad reported 10 cases in 2010, and dracunculiasis was declared endemic in Chad in 2012 after indigenous cases of dracunculiasis were confirmed over 3 consecutive years (9). Chad reported 14 cases in 10 villages in 2013, and six cases in six villages during January–June 2014. The 14 cases reported in 2013 is an increase from the 10 cases reported in 2012; and the six cases reported during January–June 2014 is increase from the five cases reported during January–June 2013. Overall, 57% of the cases reported in 2013 and 67% of the cases reported in the first 6 months of 2014 were contained. Only one of the 10 villages that reported a case in 2013 and two of the six villages that reported a case during January–June 2014 had reported a case before.

Since 2012, more dogs than humans have had emerging Guinea worms in Chad. This has not happened in any other country during the eradication campaign. Since April 2012, 49 worm specimens obtained from dogs were morphologically and or genetically confirmed to be *D. medinensis* at CDC (WHO Collaborating Center, unpublished data, 2014§). Moreover, genetic testing to compare whether the worms obtained from humans and those obtained from dogs were *D. medinensis* confirmed that they were undistinguishable (6). During November–December 2013, after five human cases (none contained) were discovered in Sarh district (Moyen Chari Region), an area under passive surveillance, The Carter Center expanded its assistance and began implementing active surveillance in that district. The working hypothesis, based on biologic, environmental, and epidemiologic investigations by CDC and The Carter Center is that the cases in humans and dogs are associated with an intense domestic and commercial fishing industry along the Chari River (where nearly all the cases have occurred) and involve a fish that serves as a paratenic host (an intermediate host in which no development of the parasite occurs). New cases occur when inadequately cooked fish are consumed by humans and when raw fish or fish entrails are consumed by dogs (6).

**What is already known on this topic?**

The number of new cases of dracunculiasis (Guinea worm disease) occurring worldwide each year has decreased from an estimated 3.5 million in 1986, when the World Health Assembly declared global elimination as a goal, to 148 in 2013.

**What is added by this report?**

The number of dracunculiasis cases reported worldwide in 2013 declined by 73% compared with 2012, and by 71% during January–June 2014 compared with January–June 2013. Transmission remains endemic in four countries, with South Sudan accounting for 70% of all reported cases during January–June 2014.

**What are the implications for public health practice?**

Although earlier target dates for global dracunculiasis eradication were missed, progress has accelerated, and eradication is likely within the next year or two if disruption of program operations caused by insecurity in Mali can be minimized.

Chad’s Guinea Worm Eradication Program and its partners continue to implement standard intervention practices in 72 priority villages at risk (Table 1). In October 2013, the program began promoting new educational messages to educate residents about proper cooking of fish and about the need to prevent dogs from eating raw fish and fish entrails. Temephos usage is constrained by the extremely large lagoons and impoundments used for fishing and as sources of drinking water. Investigations are under way to try to develop methods to isolate and treat water entry points at the end of paths leading from communities to water sources, which are routinely used by residents, and which have been identified during epidemiologic investigations as contaminated by a patient with GWD or by a dog with Guinea worm.

### Mali

Mali’s Guinea worm eradication program reported 11 indigenous cases in eight villages in 2013, an increase from the seven cases reported in 2012. Seven of the 11 cases were contained. Mali reported no cases during January–June 2014, compared with four cases (one contained) reported during the same period of 2013. In all, 85 villages were under active surveillance during January–June 2014. Cases in 2013 were reported from districts in Gao, Kidal, Mopti, and Timbuktu regions, where surveillance was still inadequate following insecurity in March 2012, although security improved somewhat in Gao, Mopti, and Timbuktu regions during January–June 2014. Médecins du Monde (Belgium) and Norwegian Church Aid are assisting the program in Kidal in 2014.

### Ethiopia

Ethiopia reported seven cases in 2013. Four of these cases were contained (one each in January, April, May, and June). The seven cases reported in 2013 were an increase from the four cases reported in 2012. After 11 consecutive months with no cases, the program reported two cases in June 2014, which is a reduction from the seven cases reported during January–June 2013. The source of both cases in 2014 is uncertain. Since October 2013, at the request of the government, The Carter Center expanded its assistance for active surveillance to include all 79 villages in Abobo district and 22 villages in Itang district, in addition to all 72 villages in Gog district, which were already under active surveillance. For several years all cases have occurred in Gambella Region, where the government and WHO have now assigned Guinea worm surveillance officers in all Guinea worm-free districts.

### Sudan

Sudan reported a small outbreak of two cases of Guinea worm disease in June 2013 and one case in September 2013. All three cases occurred at Kafia Kingi village in South Darfur, all were contained, and all patients were members of the same family. Kafia Kingi and four nearby villages at risk were placed under active surveillance and provided with health education, filters, and temephos interventions. Sudan reported no cases in January–June 2014. Dracunculiasis is not considered to be endemic in Sudan, and the country is in the precertification stage of eradication.

### Discussion

Cases reported in the global Guinea Worm Eradication Program reached a historic low in 2013, and based on current trends, fewer than 100 cases are expected to be reported in 2014. In December 2013, Nigeria was certified as free from dracunculiasis transmission—a notable milestone as Nigeria reported more cases of dracunculiasis than any other country during 1988–2008. Despite significant challenges, South Sudan’s Guinea worm eradication program is on track to eradicate the disease. The sparse, sporadic infection of humans and the unprecedented number of infected dogs in Chad are a new challenge requiring additional interventions that are currently under study.

Other challenges for governments and partners include 1) failures in surveillance and containment (e.g., missed cases, unexplained sources of cases, and uncontained cases), 2) establishment and maintenance of surveillance in dracunculiasis-free areas of all countries in which the disease still occurs or was recently eliminated, and 3) providing clean drinking water quickly to as many targeted villages as possible. Finally, insecurity in parts of Mali is now the main barrier to complete eradication of dracunculiasis.

## Figures and Tables

**TABLE 1:** Number of reported dracunculiasis cases, by country and local interventions — worldwide, 2013

	Reported cases				Villages under active surveillance in 2013				
			
Country	Indigenous in 2013	Imported in 2013^*^	Contained during 2013 (%)	Change in indigenous cases in villages under surveillance during the same period in 2012 and 2013 (%)	No.	Reporting monthly (%)	Reporting ≥1 case	Reporting only imported cases^†^	Reporting indigenous cases
South Sudan	113	0	(67)	(−78)	6,682	(100)	79	40	39
Mali	11	0	(64)	(57)	101	(85)	8	0	8
Chad	14	0	(57)	(40)	703	(100)	9	0	9
Ethiopia	7	0	(57)	(75)	72	(93)	5	1	4
Sudan	3		(100)						1
**Total**	**148**	**0**	**(66)**	**(**−**73)**	**7,558**	**(99)**	**101**	**41**	**61**

	Status of interventions in endemic villages in 2013					
	
Country	Endemic villages 2012–2013	Reporting monthly§ (%)	Filters in all households§ (%)	Using temephos§ (%)	≥1 source of safe water§ (%)	Provided health education§ (%)
South Sudan	106	(100)	(98)	(96)	(33)	(97)
Mali¶	8	(75)	(100)	(75)	(50)	(75)
Chad	2	(100)	(100)	(50)	(50)	(100)
Ethiopia	2	(100)	(100)	(100)	(100)	(100)
Sudan	0					
**Total**	**118**	**(98)**	**(98)**	**(94)**	**(36)**	**(96)**

* Imported from another country.

† Imported from another country or from another in-country disease-endemic village.

§ The denominator is the number of villages/localities where the program applied interventions during 2013–2014.

¶ Guinea Worm Eradication Program operations (supervision, surveillance, and interventions) that were interrupted in Mali’s Kidal, Gao, and Timbuktu regions as a result of insecurity beginning in April 2012, gradually improved during 2013–2014, except in Kidal region, where insecurity continues to constrain program operations.

**TABLE 2:** Number of reported indigenous dracunculiasis* cases, by country — worldwide, January 2012–June 2014

Country	2012	2013	1-yr change (%)	January–June 2013*	January–June 2014	6-month change (%)	Cases contained during January–June 2014 (%)
South Sudan	521	113	(−78)	74	19	(−74)	(79)
Mali†	7	11	(+57)	4	0	(−100)	
Chad	10	14	(+40)	5	6	(+20)	(67)
Ethiopia	4	7	(+75)	7	2	(−71)	(100)
Sudan		3		2	0	(−100)	
**Total**	**542**	**148**	**(**−**73)**	**92**	**27**	**(**−**71)**	**(78)**

* In 2012, three cases were imported into Niger from Mali and are included in Mali’s total. These persons were residents in Mali the preceding year and Niger interrupted transmission of dracunculiasis in 2008. No reports of cases imported from one country to another were reported during January–June 2014.

† Guinea Worm Eradication Program operations (supervision, surveillance, and interventions) that were interrrupted in Mali’s Kidal, Gao and Timbuktu regions as a result of insecurity beginning in April 2012, gradually improved during 2013–2014, except in Kidal region, where insecurity continues to constrain program operations.

## Footnotes

* Transmission from a patient with dracunculiasis is contained if all of the following conditions are met: 1) the infected patient is identified before or <24 hours after worm emergence; 2) the patient has not entered any water source since the worm emerged; 3) a village volunteer or other health care provider has managed the patient properly, by cleaning and bandaging the lesion until the worm has been fully removed manually and by providing health education to discourage the patient from contaminating any water source (if two or more emerging worms are present, transmission is not contained until the last worm is removed); 4) the containment process, including verification of dracunculiasis, is validated by a supervisor within 7 days of emergence of the worm; and 5) temephos is used if there is any uncertainty about contamination of sources of drinking water, or if a source of drinking water is known to have been contaminated. All of these criteria must be achieved for each emerged worm for the case to be considered contained.

† An indigenous case of dracunculiasis is defined as an infection occurring in a person exhibiting a skin lesion or lesions with emergence of one or more Guinea worms in a person who had no history of travel outside his or her residential locality during the preceding year.

§ CDC is the WHO Collaborating Center for Research, Training, and Eradication of Dracunculiasis.
